# Fabrication of endothelialized capillary-like microchannel networks using sacrificial thermoresponsive microfibers

**DOI:** 10.1088/1758-5090/ad867d

**Published:** 2024-11-19

**Authors:** John A Rector IV, Lucas McBride, Callie M Weber, Kira Grossman, Alexander Sorets, Lissa Ventura-Antunes, Isabella Holtz, Katherine Young, Matthew Schrag, Ethan S Lippmann, Leon M Bellan

**Affiliations:** 1 Department of Mechanical Engineering, Vanderbilt University, Nashville, TN, United States of America; 2 Department of Biomedical Engineering, Vanderbilt University, Nashville, TN, United States of America; 3 Department of Chemical and Biomolecular Engineering, Vanderbilt University, Nashville, TN, United States of America; 4 School of Medicine, Vanderbilt University Medical Center, Nashville, TN, United States of America; 5 Department of Cognitive Studies, Vanderbilt University, Nashville, TN, United States of America; 6 Department of Medicine, Health, and Society, Vanderbilt University, Nashville, TN, United States of America

**Keywords:** artificial capillaries, engineered microvasculature, tissue engineering, thermoresponsive polymer, top–down fabrication, microfibers, Soluplus

## Abstract

In the body, capillary beds fulfill the metabolic needs of cells by acting as the sites of diffusive transport for vital gasses and nutrients. In artificial tissues, replicating the scale and complexity of capillaries has proved challenging, especially in a three-dimensional context. In order to better develop thick artificial tissues, it will be necessary to recreate both the form and function of capillaries. Here we demonstrate a top–down method of patterning hydrogels using sacrificial templates formed from thermoresponsive microfibers whose size and architecture approach those of natural capillaries. Within the resulting microchannels, we cultured endothelial monolayers that remain viable for over three weeks and exhibited functional barrier properties. Additionally, we cultured endothelialized microchannels within hydrogels containing fibroblasts and characterized the viability of the co-cultures to demonstrate this approach’s potential when applied to cell-laden hydrogels. This method represents a step forward in the evolution of artificial tissues and a path towards producing viable capillary-scale microvasculature for engineered organs.

## 1. Introduction

The field of tissue engineering is focused on solving a diversity of medical challenges, including the growing demand for major organ transplants, the need for patient-specific medical care, and the development of more physiologically relevant platforms for drug testing [1, 2]. Meeting these challenges requires overcoming the critical hurdle of effectively vascularizing thick engineered tissues. In general, cells within living tissue can only survive a few hundred microns away from a source of vital gases and nutrients: within this range the cells’ metabolic needs are met effectively by diffusion [2–6]. Beyond this range cells begin to starve, waste products build up, and the tissue becomes necrotic. This has traditionally limited development of engineered tissues to geometries where the thickest dimension does not exceed the diffusion limit, such as with organoids and thin-walled organs [7]. In order to develop a greater diversity of artificial tissues and expand their thickness beyond a few hundred microns, a microvascular network must be engineered to carry nutrients throughout. Furthermore, to effectively support artificial tissues that may be candidate for transplantation, it will be important to also recapitulate the scale, architecture, and function of different kinds of vessels present in the human vascular tree [2, 8, 9].

Blood vessels are hierarchical and multi-functional, branching from largest to smallest and varying greatly in purpose along the way. In this hierarchy capillaries are unique: they are the smallest vessels in the body with diameters measuring roughly 10 *μ*m, they often form dense, tortuous networks, and they possess vessel walls composed of only a thin basement membrane and a single layer of endothelial cells [10, 11]. These features support the capillaries’ function as the exchange vessels for the body. They present a large surface area for solute exchange while occupying a small volume in the tissue and have vessel walls that present a small resistance to solute flux while still possessing some selective permeability. When attempting to recapitulate transport processes between the circulatory system and parenchyma, it is critical to design with the architecture of microvasculature in mind.

Techniques for engineering artificial vasculature can either be ‘bottom–up’, in which cells are instructed to self-assemble into vasculature, or ‘top–down’, in which channels are fabricated directly into a hydrogel matrix and endothelialized in subsequent steps. Bottom–up techniques have been demonstrated to produce complex, perfusable microvasculature [12–15]; however these techniques remain unsuitable for thick tissue engineering as the self-assembly of vessels requires multiple days, rendering the core of the tissue necrotic before perfusable vasculature can fully form and support it [2, 8]. Top–down approaches have been more successful for vascularizing thick engineered tissue, as the entire volume of the construct can be perfused shortly following fabrication; however, these technologies still face limitations when it comes to patterning complex microvasculature. 3D-printing of sacrificial templates has become a popular approach for top–down methods, as this technique offers programmable control over the vessel network design and flexibility in choice of materials, including hydrogel inks [16–22], carbohydrate glass [23, 24] and polyvinyl alcohol [25]. When it comes to fabricating microvessels, however, 3D-printing methods have struggled with producing networks that include channels with diameters under 100 *μ*m and network architecture with complexity in the *z*-direction. Other top–down approaches have employed optical patterning systems to produce the channels via photoablation [26], photodegradation [27–29], or photo-cross-linking [30]. While these methods offer an exceptionally fine degree of control and small feature sizes (some producing features as small as 10 *μ*m [27]) they also require expensive laser systems and optical hardware. More critically, these methods face issues when scaling up to fabricating larger tissues, including limited optical working distance and impractical amounts of time to fully pattern volumes at the centimeter-scale or larger.

Sacrificial microfibers are relatively unexplored in top–down vascular engineering outside of 3D-printed contexts. Electrospinning has been used to create microfiber meshes with diameters less than 4 *µ*m from a variety of degradable biomaterials: including natural polymers like collagen and synthetic polymers like polycaprolactone [31–33]. However, while mats of sacrificial electrospun nanofibers have been employed to form planar networks of nanochannels [34], the two-dimensional geometry of the fiber mats and the harsh chemicals used for processing limit the use of electrospinning in patterning microchannels in thick tissue constructs. Sacrificial sodium alginate microfibers with diameters ranging from 4–20 *µ*m have been produced using microfluidic platforms, but such hydrogel fibers lack the mechanical stability to create complex 3D networks prior to embedding [35, 36].

Once the channels are fabricated there remains the difficult task of culturing an endothelium on the channel walls. Modern methods of vascular engineering have made impressive strides in producing such living channels: optical techniques have created channels as small as 45 *μ*m in diameter [27] and recently an inkjet printing method produced endothelial-lined vessels with diameters of 30 *μ*m [37]. These are impressive achievements in vascular fabrication but still fall short of the scale of human capillaries. Importantly, both endothelialized networks cited above possess planar architecture and sharp, angular edges; a more biomimetic vessel network will need to possess a complexity similar to that of natural capillary beds, including tortuous channel paths, circular lumens, and 3D architecture. To date, current top–down methodologies have been unable to produce artificial endothelialized microvasculature that replicates the physiological size and architecture of human capillaries in a scalable manner.

**Figure 1.**
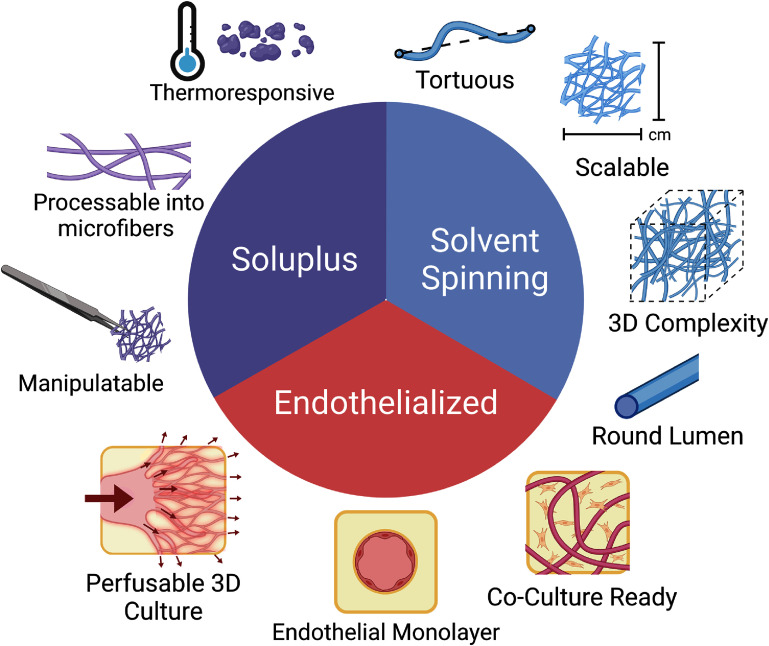
Advantages of Solvent-Spun Soluplus® Microfiber Patterning. Soluplus® is a thermoresponsive polymer which can be processed into fibers with diameters at the capillary scale, while remaining mechanical strong enough to be manipulated and positioned in tissue culture systems. The solvent spinning process is able to take this polymer and produce large quantities of microfiber meshes with a tortuous, 3D architecture and round lumens. These meshes are then embedded and removed from the system without harsh, cytotoxic processes, enabling the patterning of thick artificial tissues with endothelialized microvasculature. Illustration made using Biorender.com.

In this paper, we demonstrate a top–down sacrificial-template-patterning approach capable of producing dense, endothelialized, capillary-like channels for tissue-engineered devices. This method uses solvent spinning to process the thermoresponsive polymer Soluplus® into a sacrificial microfiber mesh, which is then employed to pattern a hydrogel matrix. This approach yields a unique combination of advantages and is used here to form microvascular networks capable of supporting thick tissue volumes seeded with cells (figure 1). We expand on the previous work by Lee *et al* [38] by seeding the channels with human umbilical vein endothelial cells (HUVECs), thereby forming an endothelium on a capillary-like microchannel network. The endothelium’s function as a barrier is demonstrated via a dextran permeability assay. Key characteristics of the vascular architecture are quantified by image analysis to demonstrate its similarity to natural capillary beds. We also demonstrate the ability to produce endothelialized microvasculature within a fibroblast-laden hydrogel and to sustain the coculture for multiple weeks. These results outline a method for producing engineered tissues possessing living vascular networks with architecture approaching that of native capillaries.

## 2. Methods

### 2.1. Cell culture

All cell types were cultured at 37 °C and 5% CO_2_ in T175 flasks according to ATCC primary cell culture protocol [39] and passaged until an appropriate population size had been achieved. HUVECs were cultured in endothelial cell media (EC media) that was formulated using Dulbecco’s modified Eagle Medium (DMEM/F12; Themo Fisher) supplemented with 5% fetal bovine serum (FBS), 10 mM L-glutamine, 0.75 U ml^−1^ Heparin, 50 *μ*g ml^−1^ ascorbic acid, 15 ng ml^−1^ IGF-1, 5 ng ml^−1^ VEGF, 5 ng ml^−1^ FGFb, 5 ng ml^−1^ EGF, 1 *μ*g ml^−1^ cortisol, and 100 U ml^−1^ pen/strep (All media supplements acquired from Sigma Aldrich or Peprotech). Human normal dermal fibroblasts (HNDFs) were cultured in DMEM supplemented with 5% FBS, 5 ng ml^−1^ FGFb, 10 mM L-glutamine, and 100 U ml^−1^ pen/strep. Media was changed every 1–2 d and cells were split and passaged into new T175 flasks upon reaching 80%–90% confluency. 0.25% Trypsin (Gibco) was used to detach cells. Cells were then spun down in a centrifuge (Sorvall Legend X1; Thermo Fisher) at 120 g for 5 min, counted and then used to seed new flasks at ∼1 million cells/flask. Cells were not used for experiments beyond the 9th passage for either cell line. Cells grown in all microchannel gels were perfused using the media employed for culture of HUVECs in flasks and media in the perfusion circuit was refreshed every 1–2 d.

### 2.2. Microfiber production

Sacrificial microfiber templates were produced by spinning a volatile solution of Soluplus® (BASF), a polyvinyl caprolactam–polyvinyl acetate–polyethylene glycol graft copolymer, out of custom rotary chamber [40–42]. The polymer solution was made by combining Soluplus® at a 50% w/v ratio with a mixture of ethanol and chloroform (7:3), and allowing it to completely dissolve over 2–3 d. To produce the fibers, 200–400 *μ*l of this solution was loaded into the chamber and spun out of side-facing ports at 3000–3200 rpm and onto a collection container (supplementary figure 1(A)). Fibers were left in a fume hood for 20 min to completely dry and then moved to a desiccation chamber until needed for device fabrication.

Microfiber production was varied by adjusting the w/v ratio of Soluplus® solution to 50%, 55%, 60%, and 65%. Motor speed was altered in each case to produce fibers with the potential for embedding in a hydrogel. Fibers were then mounted under cover slip on a microscope slide and imaged on both a widefield microscope and a stereoscope. Brightfield images of fibers were binarized and skeletonized using FIJI to extract feature values.

### 2.3. Fabrication of PDMS frames

Silicone elastomer and curing agent (Sylgard 184; Dow Corning) were mixed at a 9:1 ratio and degassed using a vacuum chamber for 10 min. A 40 × 22 × 20 mm tissue embedding mold (Thermo Scientific) was prepared by inserting three 16 G needles through the walls of the mold and across its width, such that 3 evenly spaced, parallel channels were created. Degassed PDMS was poured to a height of 1 cm in each mold and then set in an 80 °C oven for 2 h to cure. The needles and molds were then removed, producing a PDMS block with three channels. This block was cut down and used to make three individual PDMS frames that were each bonded to a thin PDMS base. The fabrication process is detailed in supplementary figure 2 with visual aids.

### 2.4. Fabrication of microchannel gels

To establish a point of fluidic connection, a 1/16”, polypropylene, luer-to-barb fitting (Cole-Parmer) was inserted into a circular port on the previously fabricated PDMS frames. A length of 1/32” PTFE tubing (Cole-Parmer) was then inserted into both the luer-lok connector and the opposite port in the PDMS, so that about 1 mm of tubing was protruding into the frame to form a large-inlet channel. A layer of Soluplus® was then lightly painted onto the exposed tubing and side-walls of the PDMS device using a thin wooden dowel coated in the Soluplus® solution. A small section of Soluplus® microfibers was then cut and inserted into the frame, ensuring that connections were established between the fiber cluster and the regions painted with Soluplus®. Fiber-laden frames were prepared in batches and allowed to dry for at least 20 min in a fume hood. In groups of three, fiber-laden frames were plasma treated in a Harrick Plasma Cleaner on high power at 700–800 mTorr for 20 min to ensure sterility and increase wettability of fiber surfaces. Immediately after plasma treatment, devices were moved to a biohood. Sterile solutions of 0.1 g ml^−1^ gelatin (Sigma-Aldrich; gel strength 300, Type A) in EC-media and 0.1 g ml^−1^ microbial transglutaminase (mTG; #1203-50, Modernist Pantry) in 1 × PBS were kept at 40 °C in a water bath. The two solutions were mixed at a 9:1 ratio until homogeneous and then immediately dispensed into the plasma treated, fiber-laden frames. For fibroblast co-culture experiments, a 0.12 g ml^−1^ gelatin solution, a suspension of 1 × 10^7^ fibroblasts ml^−1^, and a 0.1 g ml^−1^ mTG solution were combined at a ratio of 8:1:1 and then dispensed into frames. For permeability assays, gels were prepared with a 0.05 g ml^−1^ solution of gelatin in order to make any observable changes more pronounced. Gels were then moved into a cell culture incubator to crosslink overnight. Once fully crosslinked, devices were removed from incubation and allowed to cool to room temperature inside a biohood (∼5 min), causing the Soluplus® to undergo a gel-to-sol transition as it cooled below its lower critical solution temperature (LCST). Devices were then perfused with 200 *μ*l of EC media to clear liquified Soluplus® from the channels. This was performed using a 200 *μ*l micropipette: the tip was pressed into the barb of the device and gentle pressure applied to drive flow through the channels. Devices were then returned to the incubator with a layer of media on the gel surface to prevent dehydration until seeding.

### 2.5. Microchannel seeding

HUVECs were cultured in flasks until 70%–90% confluent and then detached, spun down and resuspended in fresh EC media at a concentration of 1 × 10^7^ cells ml^−1^. For each microchannel gel, a 200 *μ*l pipette tip was loaded with 100 *μ*l of cell suspension and inserted into the barb such that a seal was formed between the pipette tip and barb inner wall. Steady pressure was then applied to inject the cell suspension into the barb and large inlet-channel of the device, allowing it to flow into the channels without rupturing the gel. The full 100 *μ*l was injected over 1–2 min. Devices were placed into individual petri dishes and returned to the incubator to allow cells to adhere for 15 min. Devices were then taken from the incubator, flipped over in their petri dish and returned to the incubator for an additional 15 min, so that cells adhere to the other half of the inlet-channel. Following this, devices were oriented such that the luer-lok inlet was facing downward and then placed into a 50 ml conical tube of media to allow remaining unattached cells in the inlet-channel to fall out of the device; this step was needed to avoid cell aggregates clogging the microchannel network. Conical tubes containing devices were incubated for another 2 h before devices were then removed and attached to perfusion circuits.

### 2.6. Microvascular gel perfusion

To determine the connectivity of microchannel networks, they were perfused with 1 mg ml^−1^ Rhodamine B (#132315000, Thermo Fisher Scientific) in 1 × PBS using a syringe pump (New Era Pump Systems). A low magnification video of the dye reaching different parts of the microchannel gel was taken. Microchannels were also perfused with a dispersion of two colors of fluorescent microbeads to collect microscopic video of flow in different regions of the microchannel gels. This dispersion was prepared using a 1:100 dilution of 0.02 *μ*m, red FluoSpheres (#F8786, Invitrogen) and a 1:2000 dilution of 2 *μ*m, green Fluospheres in 1 × PBS. The smaller microbeads appeared as a homogeneous fluorescent liquid and highlighted the volume of the microchannels, while the larger microbeads appeared as discrete particles that could be tracked flowing through the channels. Particle tracking was performed in FIJI in order to extract estimations for flow rates in channels.

For extended culture of endothelial cells in microchannels, a perfusion circuit was adapted from the work of O’Grady *et al* [43] to pump fresh media into the gel during incubation. The perfusion circuit consisted of a deep-welled petri dish containing a bath of EC media, a microchannel gel in its PDMS mold, and gas-permeable tubing. A custom-built peristaltic pump drove media from the media bath, through the tubing circuit, and into the microchannel gel, which rested in the same media bath. Calibrations were performed to relate the pump motor speed setting to the time-averaged flow rate at the inlet.

### 2.7. Channel architecture characterization

In order to visualize the channel architecture of the microvascular gels, channels were perfused with a 2.5% w/v gelatin + mTG (9:1 ratio) solution that was prepared with a 1:10 dilution of 0.04 *μ*m FluoSpheres with AlexaFluor-488 labels (#F8845, Invitrogen). Both the hydrogel devices and the fluorescent bead-gel solution were kept at 40 °C prior to perfusion. Gels were then incubated for 1 h to allow the bead-laden gel to crosslink in channels. Gels were then removed from their PDMS frames and imaged with a Zeiss Z1 Lightsheet microscope. Image stacks were acquired in the *z*-direction with 50% overlap between images to optimize data collection. Multiple regions of interest were imaged from each device to gain a representative set of channel architecture data. Image stacks were filtered, resized and thresholded into binary matrices using FIJI. These image stacks were then fed into a MATLAB script, where the binary matrices were skeletonized and the resulting architecture characterized. This MATLAB script was modified from its original version written to characterize vasculogenesis in iPSC derived endothelial cultures [44–46].

### 2.8. Immunohistochemistry

Cell-laden microvascular gels were perfused with 4% paraformaldehyde (PFA; #J19943.K2, ThermoFisher Scientific) for 5 min at 50 *μ*l min^−1^ and then immersed in 4% PFA for an additional 5 min. Devices were then perfused with 0.1% Triton-X (#A16046.AE, ThermoFisher Scientific) in 5% goat serum (#50062Z, Life Technologies) to permeabilize cells. Devices were then perfused with pure 5% goat serum for 10 min at 50 *μ*l min^−1^ to clear out remaining triton and PFA before being submerged in 5% goat serum for 48 h at 4 °C to block non-specific binding sites.

HUVECs were stained with 6 *μ*M Hoechst 33 342 (#62249, Thermo Scientific) to identify nuclei and a 1:20 dilution of phalloidin conjugated to AlexaFluor-488 (#A12379, Invitrogen) to identify actin, thereby highlighting the entirety of cell bodies. Solutions of Hoechst and phalloidin were perfused into devices at 50 *μ*l min^−1^ for 20 min to stain cells. In some devices, cells were also labeled with AlexaFluor-647 conjugated primary antibodies to target specific surface proteins (VE-Cadherin: #ab272346, Abcam; PECAM-1: #49940 S, Cell Signaling Technology). For both antibodies, 100 *μ*l of a 1:10 dilution of antibody in blocking buffer was injected into the device and submerged in just enough blocking buffer to cover the gel in a 50 ml centrifuge tube. Tubes were wrapped in tin foil and devices allowed to incubate at 4 °C for 48 h with antibody. Following this, stained gels were removed from their PDMS frames and incubated for 24 h on a rocking plate at room temperature in 1 × PBS with 0.02% sodium azide (#014314; ThermoFisher Scientific) to clear blocking buffer and unbound stain from the gel.

Mouse brain tissue clearing and staining is described in Supplemental Information.

### 2.9. Dextran permeability assay

Barrier functionality of the endothelial monolayers was evaluated by dextran permeability assay. Assays were performed on both acellular (nude) channels and endothelialized channels (cultured for 7 d) to compare the permeability of channel walls with and without an endothelial monolayer. All devices were connected via silicon tubing to a syringe pump (NE-300; New Era Syringe Pumps) and imaged through a glass bottom petri dish (#P35G-1.5-14-C; MatTek). For each device, a region of interest was located prior to dextran perfusion, to minimize the time between dextran first reaching the region and the beginning of imaging. Channel regions that were horizontally oriented and isolated from other neighboring channels were identified so that a wide, one-dimensional diffusion profile could be captured. Prior to removal from perfusion circuits, endothelialized channels were perfused for 20 min with a 5 *µ*M solution of Calcein Red AM (21900; AAT Bioquest) to enable visualization of the HUVECs and ensure complete cell coverage of the region of interest. At time zero, channels were perfused with a 0.5 mg ml^−1^ solution of 500 kDa Dextran-FITC (#FD500S; Millipore-Sigma) in DMEM at 10 *μ*l min^−1^. Confocal microscopy images were acquired every 1 min for at least 30 min. Imaging was started prior to dextran reaching the channel to capture the early, and most critical, time points. Time series images were registered in FIJI to eliminate any artifacts due to drift. Permeability values were then extracted from the time series using a MATLAB script written by Polachek *et al* [47] (our imaging methods closely matched those used in this paper).

### 2.10. Propidium iodide (PI)-DAPI viability assay

We found that a traditional Calcein AM live stain made individual cell bodies very difficult to quantify, especially in the case of contiguous endothelial monolayers. To circumvent this issue, we used a multi-step staining and imaging process which used PI to quantify the dead cells and DAPI to quantify every cell, and then used these values to derive a percent of viable cells.

EC-HNDF co-culture microvascular gels were removed from their PDMS frames and cut in half using a fresh razor blade. These halves were submerged in a combination of 5 *μ*M Calcein AM (# C3100MP, Invitrogen) and 4 *μ*g ml^−1^ PI (#P3566, Life Technologies) in 1 × PBS and incubated for 20 min. Gel halves were then rinsed twice using warm PBS and placed into a 24-well plate such that the cut-side could be imaged. Dead cells appeared as red fluorescent puncti. Multiple *z*-stacks of 100 *μ*m thickness were acquired with a 10x objective for each gel. To ensure that cells damaged from the cutting were not included, image acquisition began no less than 200 *μ*m into the gel. Following imaging of Calcein + PI-stained gels, the samples were fixed in a bath of room-temperature 4% PFA for 10 min. Fixed samples were rinsed with 1 × PBS and then transferred to a bath of 0.1% Triton-X and agitated on a rocker plate for 1 h. Samples were washed once again in 1 × PBS and allowed to sit overnight in PBS + 0.02% sodium azide. Samples were then stained with 1 *μ*g ml^−1^ DAPI for 30 min on a rocker plate at room temperature. Samples in wells were finally rinsed twice with 1 × PBS and then submerged 1 × PBS + 0.02% sodium azide until ready for imaging. The same region of interest was identified and matched to previous image sets by aligning the channel features visible by using the pseudo-brightfield image produced by the microscope’s TPMT. These ROIs were imaged again, this time capturing the blue, fluorescent nuclei of all cells.

Using FIJI, *z*-stacks were flattened into *z*-projections. The red channel from a PI image stack and the blue channel from a DAPI image stack of the same sample were then co-registered to each other to ensure the same sample volume was used for quantification. Automated cell counts were performed, totaling the number of blue and red particles. With the total number of cells and the total number of dead cells, a percentage of viable cells was calculated for each ROI.

### 2.11. Imaging and image processing of endothelialized microchannels

Brightfield imaging was performed using a Zeiss Z1 Observer. Lightsheet imaging was performed using a Zeiss Z1 Lightsheet. Confocal imaging was performed with a Zeiss LSM710 confocal microscope. Images were taken using an array of Zeiss objectives: Fluar 5×/0.25, EC-Plan Neofluar 10×/0.3, LD-Plan Neofluar 20×/0.4, and Plan-Apochromat 20×/0.8. Projections of *z*-stacks, image overlays, and composites were created using the freely available image processing software ImageJ and software packages included in the ImageJ expansion, FIJI.

## 3. Results and discussion

### 3.1. Fabrication of the artificial microvascular hydrogels

Fabrication of the artificial microvascular devices began with the creation of sacrificial microfiber meshes. We needed a material that was non-toxic, soluble in both aqueous and volatile organic solvents, and could be removed from the channels without disturbing the hydrogel matrix. We began by testing multiple thermoresponsive polymers, including Pluronic F-127, poly(N-isopropylacrylamide), polyvinylcaprolactam, and Soluplus®, a polyvinyl caprolactam–polyvinyl acetate–polyethylene glycol graft copolymer. Of these four, Soluplus® performed best: it produced the most mechanically robust microfibers, demonstrated negligible toxicity to cell cultures, and most importantly, showed temperature-dependent solubility in water [40–42]. At high concentration, Soluplus® exhibits a LCST-like behavior at 32–37 °C: below this temperature it becomes more favorable for Soluplus® to adopt an unfolded configuration, form hydrogen bonds, and become much more soluble in water [41, 48]. By exploiting these properties, we were able to produce sacrificial microfiber templates that could maintain their geometry at physiological temperatures and then dissolve when cooled to room temperature.

Although this method of template fabrication forfeits fine control to gain scalability and three-dimensional complexity, fiber production can still be qualitatively tuned to produce variable features. The factor that made the highest impact on the resulting fiber features was the weight/volume percentage of Soluplus® in the volatile spinning solution. By varying the w/v% from 50%–65% and then adjusting the motor speed to produce viable fibers, we were able obtain a range of median fiber diameters from 12.8 *µ*m to 26.8 *µ*m and median branch lengths from 45.6 *µ*m to 88.1 *µ*m (supplementary figure 3, table S1).

**Figure 2.**
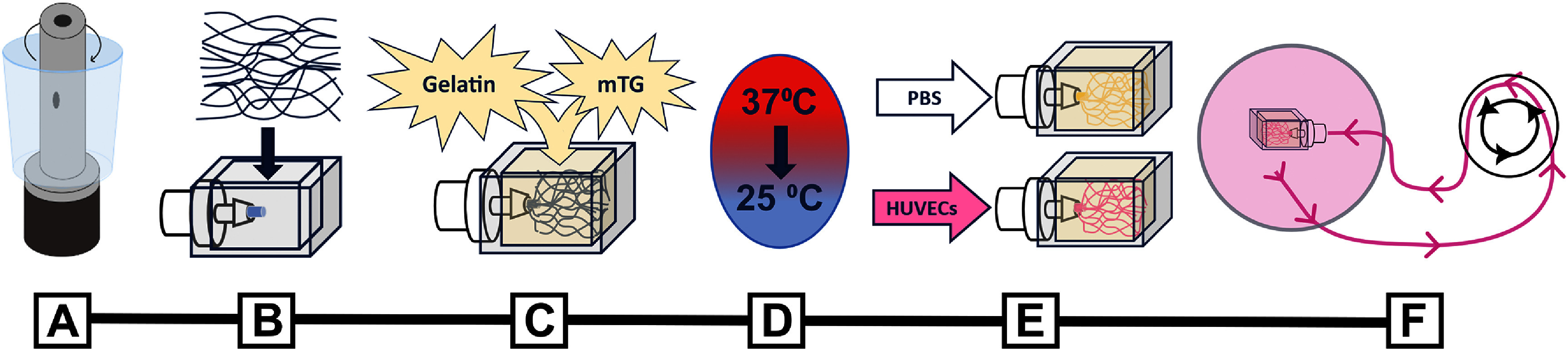
Schematic of fabrication process for microvascularized hydrogel devices. (A) Microfibers are solvent spun, (B) loaded into PDMS frames, (C) embedded in mTG-gelatin, (D) cooled below LCST to dissolve, and (E) flushed out of crosslinked hydrogel. Microvascularized hydrogels can then be (E) seeded with ECs and (F) attached to perfusion.

Soluplus® fibers were spun and embedded into a gelatin-mTG matrix held within a PDMS frame (figures 2(A)–(C); supplementary figure 1). These hydrogel devices were cooled to room temperature to trigger the liquification of the hydrated Soluplus® microfibers (figure 2(D)). This method successfully and repeatably produced dense microchannel networks, extending in three-dimensions in their hydrogels (figure 2(E)).

We perfused microchannel gels with Rhodamine B and found that the channels were highly interconnected with dye visible throughout the branching network and extending from inlet to outlet (supplementary video 1). Perfusion of channels with a dispersion of fluorescent microbeads allowed us to both confirm the connectivity of our channel network and characterize the range of flow rates present in the microchannels (supplementary figure 1(E); supplementary videos 2–4). For an inlet flow rate of 25 *µ*l min^−1^ across 4 microchannel gels, we found that the middle 80% of flow rates in channels ranged from 0.32–4.70 mm s^−1^ (*n* = 28). Individual flow rates were also used to calculate the shear stress experienced by cells on a channel wall; the middle 80% of this ranged from 0.03 to 0.32 Pa (*n* = 28). Natural capillaries have flow rates typically ranging from about 0.5–1.5 mm s^−1^ and experience internal shear stresses from about 1–2 Pa [49–53]. Comparing the values from our microchannel gels to natural capillaries, we are achieving flow rates of similar order of magnitude, and exposing channels to shear forces far below levels at which endothelial barrier function begins deteriorating (4 Pa) [51].

### 3.2. Characterization of artificial microvascular architecture

In order to capture the architecture of our artificial microvessels, devices were perfused with a suspension of fluorescent microbeads in a thin gelatin-mTG solution. This allowed the fluorescent solution to be perfused through all fluidically viable channels and then solidify to retain the channels’ shape. We employed 40 nm Fluorospheres because they appear as a homogeneous fluorescent region at lower magnification and diffused slowly enough that they would show well-defined channels walls if imaged on the day of perfusion.

**Figure 3.**
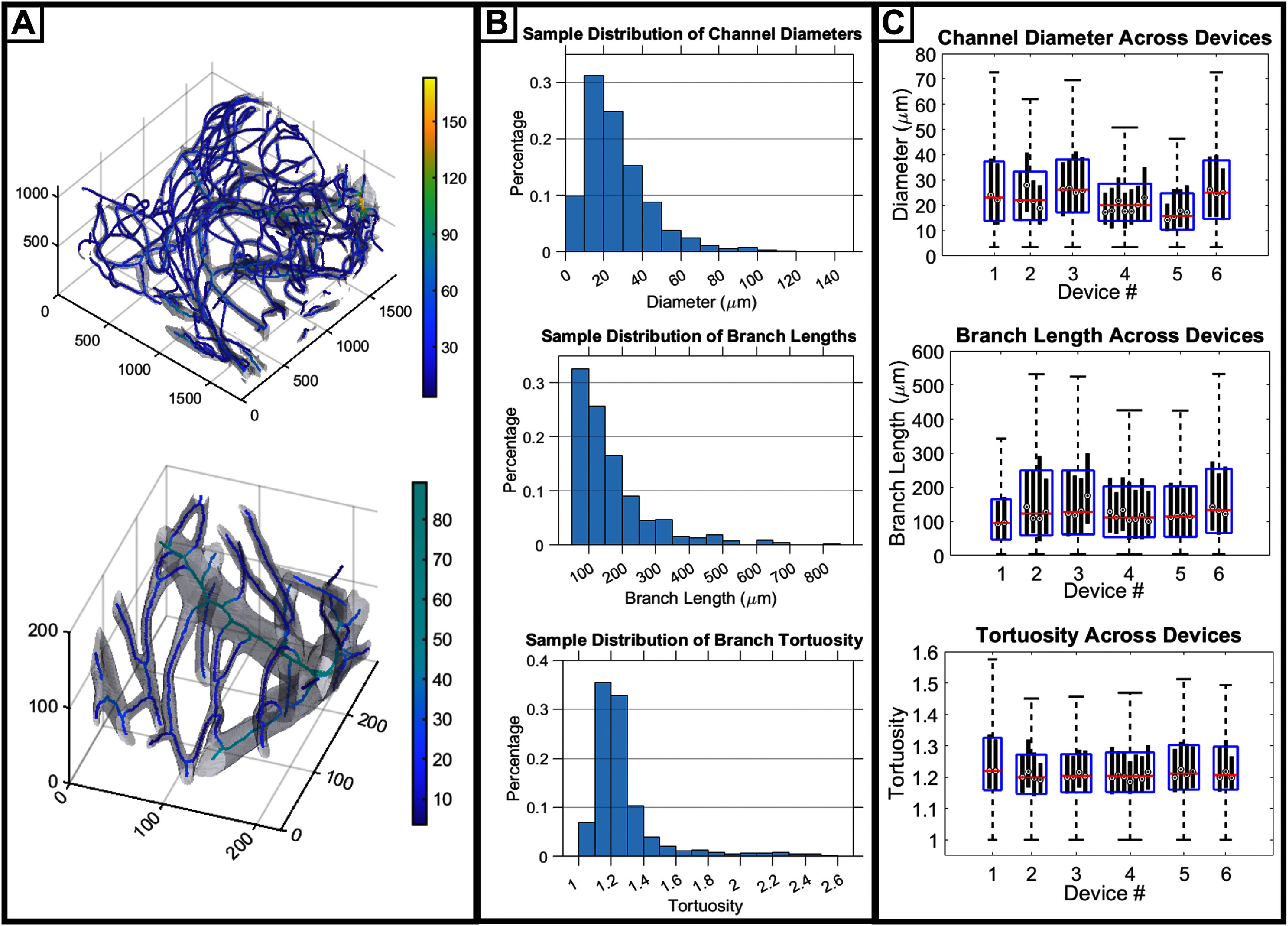
Characterization of the engineered microvascular architecture. (A) A skeletonized microchannel network with entire channel volume overlayed in grey. The heatmap displays the diameter of the channel associated with each skeleton voxel. Units for all axes and heatbars are in *μ*m. (B) Sample histograms of architectural features extracted from one ROI in a single device. The diameter histogram shows a distribution of skeleton voxels, and the tortuosity and branch length histograms show a distribution of branches. (C) Box and whisker plots of architectural features extracted from multiple devices. Black bars show the interquartiles of individual regions of interest, with circles showing the median. Blue rectangles show the interquartiles for the compiled data of all regions of interest in each device, with red line showing the median and whiskers showing 1.5× the interquartile range, containing approximately 95% of data points.

Channels filled with crosslinked fluorescent gelatin solution were imaged via lightsheet microscopy. Multiple regions of interest were captured per device as *z*-stacks with dimensions of approximately 1.7 mm length and width by 0.5–1.5 mm depth. We found that 3D reconstructions of the *z*-stacks bore a strong resemblance to human microvasculature [54]. After processing, image stacks were inspected by manually measuring and comparing the channel diameters from pre and post processed images: if the comparison showed an error of less than 10% or 4 *μ*m, then it was approved for architecture characterization. Visual inspection of skeletonized channels shows complete and connected skeleton networks that faithfully follow channel architecture (figure 3(A)).

To quantify microchannel network architecture, we extracted channel diameter, branch length, tortuosity, channel volume fraction, and the portion of the gel within 200 *µ*m of a channel from each dataset (table 1). Channel diameter is a key feature to evaluate our architecture, as it indicates the scale of features that this method can produce. Branch length shows the average length of a vessel segment between two branch points, which helps define the degree of connectivity within the network. Tortuosity is calculated here as the total path length of a vessel segment divided by the linear distance between its two endpoints. The tortuosity gives a point of reference for how much the paths of the artificial vessels resemble those taken by natural blood vessels. Channel volume fraction tells us how much space is occupied by the vessel network and the portion of the gel within 200 *µ*m of a channel tells us what percentage of the ROI exists within a range that could be supported by diffusion from the channel network. Together, these features help characterize the density of the channels in the gel and give an approximation of how much surrounding tissue this network could support in a cell-dense environment. Feature values were computed for each individual ROI of a microchannel gel and then averaged together to produce a mean value representative of the whole sample. The ‘whole sample means’ were then averaged together to produce a final value representing the average feature value across all samples (*n* = 6). Histograms of channel features show that most feature values are distributed lower than the mean, therefore we also include the median to better represent the most common value for each (figure 3(B), table 1). Standard deviations for each feature were calculated as both intra-device, or the standard deviation between ROIs in a single device, and inter-device, or the standard deviation between different devices. It is worth noting that not all the same image stacks were suitable for extracting density features, as some also captured large regions devoid of channels that would be inappropriate to include in the ROI for volumetric calculations. Because of this, density features were quantified using a subset of image stacks that described volumes bound by channels on all 6 sides (*n* = 5). As a point of comparison to our artificial architecture, lightsheet microscopy data obtained from capillary beds of mouse brain cortex were processed using the same scripts in FIJI and MATLAB to obtain the same features for natural tissue (table 1, *n* = 6). In choosing this we considered that mice are extensively used as neurovascular models for disease [55]. Others have also found that murine and human cerebral capillaries possess topologically similar anatomical networks, and differ in geometric scale by a factor of less than 20% [56]. Considering these factors, we believe that mouse cortical capillaries present a reasonable target for comparison.

**Table 1.** Feature quantification of engineered microvascular architecture.

Feature	Median	Average	Intra-device stddev	Inter-device stddev	Mouse cortex
Channel Diameter (*µ*m)	21	26	±17	±4	7.3 ± 2.8
Branch Length (*µ*m)	112	214	±180	±32	98 ± 71
Tortuosity (1)	1.21	1.32	±0.36	±0.03	1.35 ± 0.36
Channel Volume Fraction (%)	2.0	3.0	±0.05	±1.6	2.5 ± 0.9
Portion of Gel within 200 *µ*m of a Channel (%)	69	78	±4.8	±10	100 ± 0

Features extracted from the 6 microchannel gels describe a complex network of microvascular-like channels. Overall, the artificial vessels fabricated from microfiber templating possessed an average diameter of 26 ± 17 *μ*m and a median of 21 *μ*m. This represents a smaller scale of feature than is currently being created by contemporary templating techniques and can be done at larger scale than optical techniques. Looking at the distribution of channel diameter from a single ROI, we can also see the presence of channels with diameters on the scale of human capillaries, with about 10% of skeleton voxels associated with a channel diameter ⩽10 *μ*m (figure 3(B)). It is worth noting that our method was able to produce channel networks with a higher percentage of ⩽10 *μ*m channels; however, we chose to embed fiber meshes with slightly larger average diameters in order to better facilitate EC seeding. Additionally, the average tortuosity of vessel branches, 1.32 ± 0.36, matches remarkably closely with that of the mouse cortex capillaries, 1.39 ± 0.42. This indicates that the paths of the individual artificial vessel branches closely resemble those taken by biological capillaries. The average artificial channel branch length had a mean of 214 ± 180 *μ*m and a median of 112 *μ*m; this average is close to the average measured in mouse brain capillaries, 145 ± 90 *μ*m, and while the standard deviation is somewhat large, it still shows similarity within an order of magnitude. Channel density is perhaps the feature most in need of improvement: despite having an average diameter 4× larger, the mouse capillaries still occupy a larger average volume fraction, indicating the presence of a much larger total capillary length per tissue volume. That said, with the objective of eventually supporting cell-dense engineered tissues, having nearly 80% of the ROI’s volume within the 200 *μ*m diffusion limit is a promising result. Since the density of channels in a hydrogel is currently controlled by manual packing of fiber meshes, this is a feature that can be theoretically improved with few changes to the method.

Despite the microfiber templates forming chaotically via solvent spinning, there was a high degree of similarity between features from each device. Box and whisker plots of diameter, branch length, and tortuosity show not only a tight grouping of regions of interest within each device (indicated by the solid, vertical, black bars) but a tight grouping of the medians between all devices (indicated by the horizontal red bars) (figure 3(C)). This is supported by the small inter-device standard deviations, several of which were smaller than the average intra-device standard deviation. This indicates a high degree of repeatability for the patterning technique, despite taking advantage of a chaotic fabrication process.

### 3.3. Endothelialization of artificial microvascular channels

**Figure 4.**
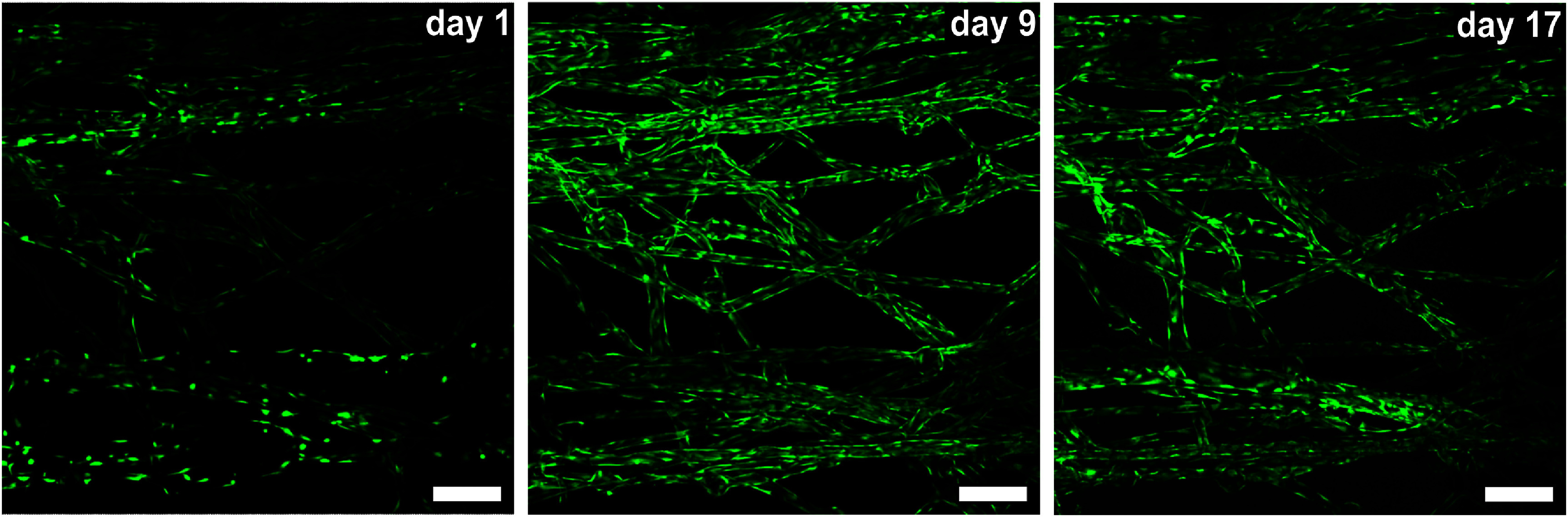
Montage of HUVEC-lined channels, stained with Calcein AM. HUVECs spreading in channels at days 1, 9, and 17 post-seeding. Scale bars are 250 *μ*m.

**Figure 5.**
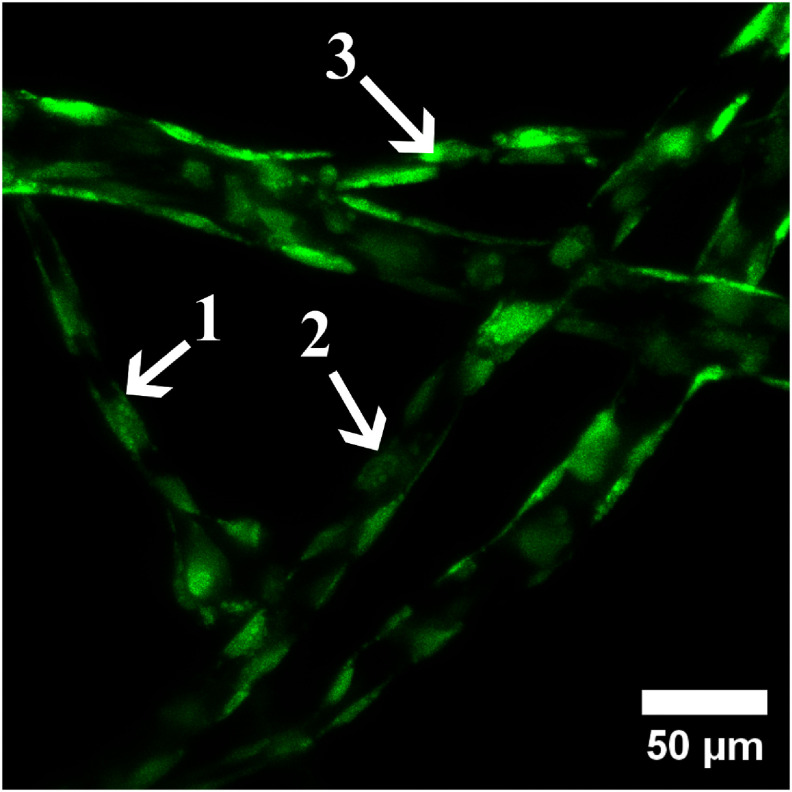
HUVEC-lined channels, stained with Calcein AM and imaged with 20× objective. Channels imaged at day 21 post-seeding. Arrows indicate channels with diameters of (1) 12.6 *μ*m, (2) 19.8 *μ*m, and (3) 14.2 *μ*m.

HUVECs were seeded into the artificial microchannels to form a contiguous endothelial monolayer. To assess EC viability and observe cell behavior, endothelialized devices were regularly perfused with a 5 *μ*M Calcein AM and imaged via confocal microscopy. Viable HUVECs were observed to adhere and spread over the first 24 h; following this, they were observed proliferating, migrating, and joining together to form a monolayer around the circumference of the channel lumen (figure 4, supplementary figure 4, supplementary video 5). EC monolayers in artificial microchannels could regularly be maintained and remain viable for over 3 weeks. In addition, HUVECs cultured in our artificial microchannels were found occupying channels smaller than 13 *μ*m in diameter (figure 5). Contemporary microvascular engineering has demonstrated individual, isolated, endothelial-lined straight channels with diameters approaching those of capillaries, such as those produced by Zhao *et al*’s double templating method [57]. Similarly in 2021, Zheng *et al* applied electrohydrodynamic inkjet printing to reduce the scale of their endothelialized, 3D bioprinted channels to as small as 30 *μ*m. While these publications present some of the smallest, live, artificial vessels to date, the geometry of their channels remain limited to either individual segments of to a single plane of construction. The data presented here show that our thermoresponsive templating technique is capable of producing living channels that not only approach the scale of natural capillaries, but also posses a three-dimansional, interconnected, and tortuous architecture.

**Figure 6.**
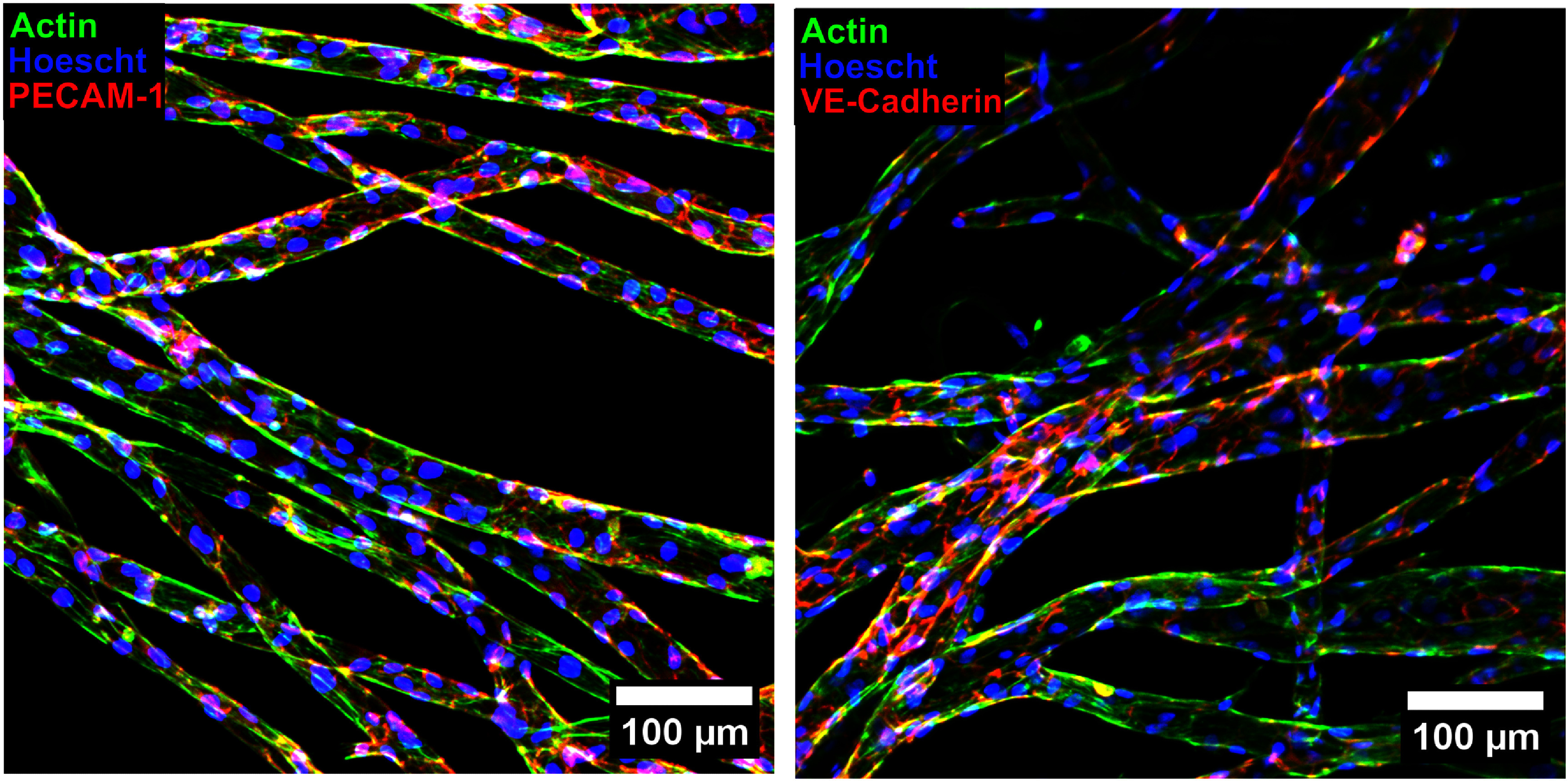
IHC stained HUVECs lining artificial microvessels. Devices were fixed at day 14 post-seeding. For both images, blue signal shows cell nuclei and green signal shows actin. The red signal shows the presence of PECAM-1 (left) and VE-Cadherin (right).

To further evaluate the health and functionality of the EC monolayers, IHC staining was performed at 14 d post-seeding (figure 6, supplementary video 6). Images of HUVECs stained for phalloidin support our previous assertions that HUVECs are proliferating in channels after seeding to cover channel surfaces and form a contiguous monolayer (supplementary figure 5, supplementary videos 7 and 8). Additionally, a cross-section of an IHC stained channel confirms full circumferential coverage of the channel wall (supplementary figure 6). The cell-to-cell junction proteins PECAM-1 and VE-Cadherin were both observed at endothelial junctions across the cell-covered channels (supplementary figure 7). The presence of these junction proteins indicates formation of a healthy EC monolayer.

### 3.4. Endothelial barrier functionality

**Figure 7.**
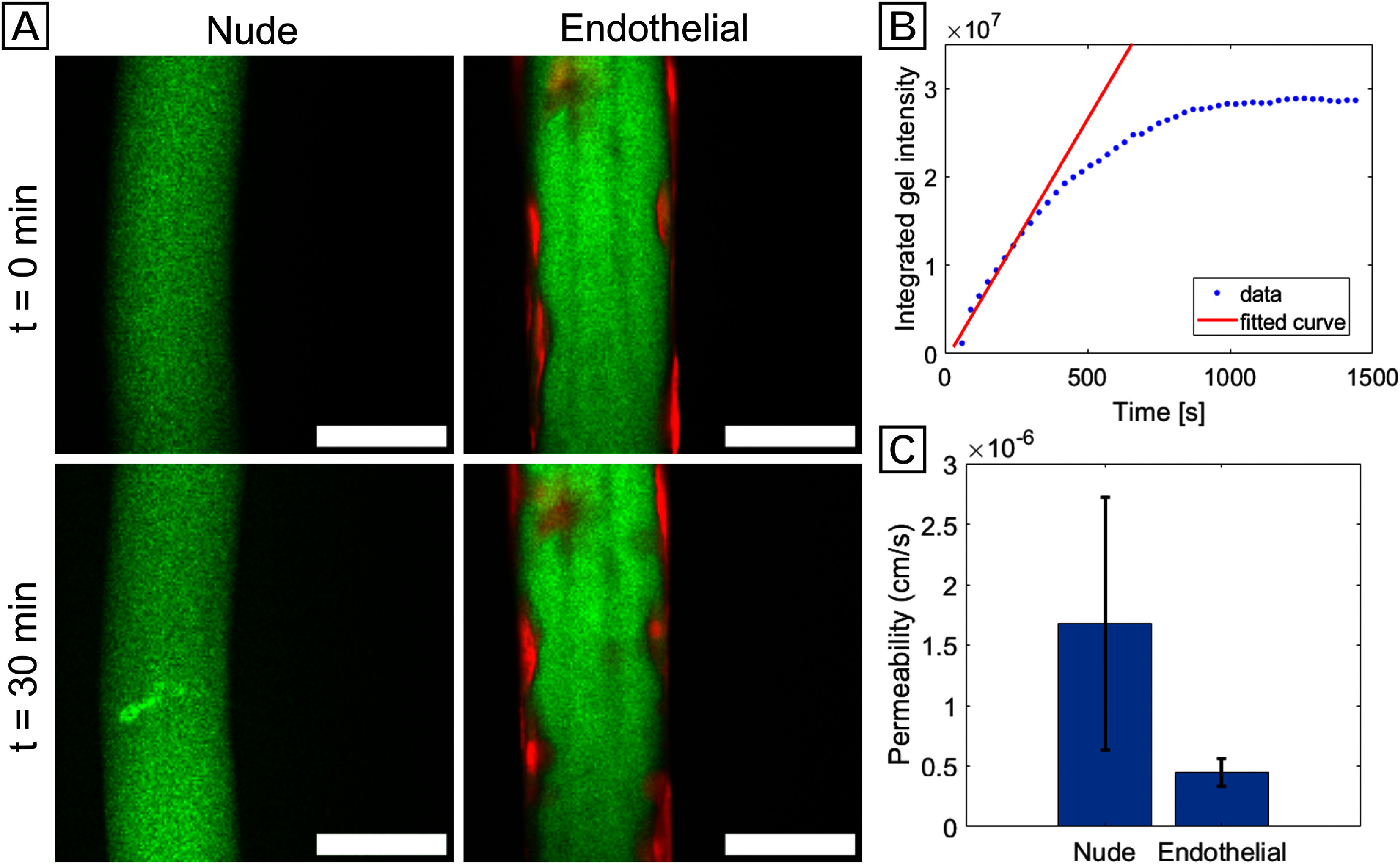
Permeability artificial microchannels to 500 kDa dextran-FITC. (A) Diffusion of 500 kDa dextran-FITC from nude and endothelialized microchannels over 30 min. 500 kDa dextran-FITC is shown as green and HUVECs labeled with Calcein Red AM are shown as red. Scale bars in all images are 50 *µ*m. (B) Example plot of integrated fluorescence intensity within the gel surrounding the channel over time. Curve fits were based on early time points to approximate a linear relationship between intensity and time. (C) Permeability of nude channels versus that of channels with an endothelial lining.

An important function of the vascular endothelium is to create a selectively permeable barrier between the bloodstream and tissue; therefore, we evaluated the ability of our endothelialized microchannels to create such a barrier. This was done using a dextran permeability assay, in which a solution of 500 kDa dextran-FITC was perfused into the microchannels and observed diffusing through the channel wall into the surrounding gel matrix. (figure 7(A)). The fluorescence intensity for both regions were integrated for each point in a time series and used to calculate permeability with equation (1), based on a derivation of Fick’s First Law where *P* is the permeability, *r* is the radius of the channel, *I*_channel_ is the constant integrated fluorescence intensity inside the channel, and d*I*_gel_*/*d*t* is the change in the fluorescence intensity of the surrounding gel over time [47, 58],

\begin{align*}P = \frac{{2r}}{{{I_{{\text{channel}}}}}}*\frac{{{\text{d}}{I_{{\text{gel}}}}}}{{{\text{d}}t}}\end{align*}

For this analysis to be valid, it is assumed that the fluorescence intensity is directly proportional to the concentration of fluorophore, that the thickness of the optical slice imaged is much less than the radius of the vessel, and that the concentration of fluorophore in the gelatin matrix is negligible compared to that inside the channel (true at early timepoints). With these assumptions in place, a linear fit can be applied to the relevant window of dextran diffusion and a value for d*I*_gel_*/*d*t* can be extracted (figure 7(B)). Values for the remaining variables are simple to measure and the permeability is straightforward to calculate.

Microchannels with HUVEC cultures at day 7 showed increased barrier functionality for 500 kDa FITC-dextran as compared to nude microchannels. Qualitative comparison of confocal images shows a brighter hydrogel region near the nude channels after 30 min of perfusion compared to the endothelialized channel gels (figure 7(A)). This shows a higher concentration of dextran diffusing out of the channel over the same period, and thus a higher permeability when no endothelium is present. Quantification of permeability values for each channel wall type confirms this, as the average permeabilities for nude and endothelialized channels were 1.7 ± 1.0 × 10^−6^ cm s^−1^ and 0.4 ± 0.1 × 10^−6^ cm s^−1^ respectively (figure 7(C)). Welch’s t-test performed on the two groups of permeability data yielded a *p*-value of 0.003. The endothelium cultured in our patterned microchannels significantly reduces the permeability of the channel wall to 500 kDa dextran, and thus possesses barrier properties characteristic of a functional capillary wall.

### 3.5. Microvascularized fibroblast co-culture

**Figure 8.**
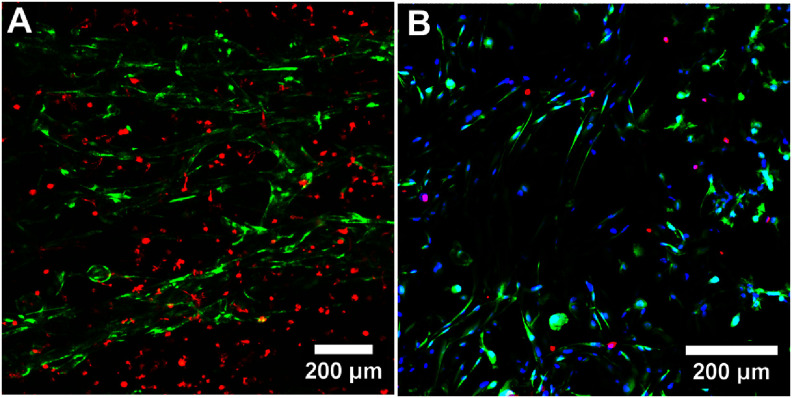
Cocultures of HUVECs and HNDFs in microvascular hydrogel devices. (A) GFP-HUVECs (green) localized to microchannels and RFP-HNDFs (red) to gelatin matrix. Image collected at day 7 post-seeding. (B) Viability assay of HUVEC-HNDF coculture at day 7 post-seeding. Image shows an overlay of live-dead stain taken pre-fixation and DAPI stain taken post-fixation for a single region of interest: PI (red) shows dead cells, Calcein AM (green) shows live, and DAPI (blue) shows nuclei of all cells.

For our microchannel fabrication method to have broad, practical application, it will need to be able to produce vascular structures within cell-laden hydrogels. To demonstrate this, we patterned HUVEC-lined microchannels in gelatin hydrogels seeded with HNDFs. Fibroblasts were employed as the cell type to seed in the matrix because they are a common cell found throughout the parenchyma of the body and have previously been used to demonstrate the utility of microvasculature within a gel [38, 59]. Our artificial microchannel gels were prepared identically to previously described methods, but with the addition (during the fiber embedding step indicated in figure 3(C)) of HNDFs to the gelatin-mTG mixture to a final concentration of 10^6^ cells ml^−1^. Channels were then cleared, seeded with HUVECs, and connected to peristaltic perfusion as previously described. After a week of culturing, confocal images of green fluorescent protein expressing HUVECs (GFP-HUVECs) and red fluorescent protein expressing HNDFs (RFP-HNDFs) showed well defined niches for each cell type, with GFP-HUVECs spreading throughout microchannels and RFP-HNDFs well dispersed in the gel matrix (figure 8(A)).

After two weeks of culture, HUVEC-HNDF cocultures were assayed for cell viability. A traditional live-dead assay uses Calcein AM to stain for viable cells; however, we found that individual cell bodies were very difficult to quantify as the entire cell body was stained and would frequently overlap with a neighboring cell or with cells in image layers above or below: this was especially true in the case of contiguous endothelial monolayers in microchannels. To circumvent this issue, we used a multi-step staining and imaging process which used PI to quantify the dead cells and then DAPI to quantify total cells (figure 8(B), supplementary figure 8). Using these two values, a percentage of living cells could be easily calculated. Co-culture gels were cut in half so that viability could be assessed for cells directly supported by nearby channels. After 7 d post-seeding HUVECs and HNDFs in 3D microchannel co-cultures collectively showed a viability of 90 ± 2% (*n* = 6), with fibroblasts displaying spreading behavior (figure 8(B)). The distinct viabilities of endothelial and fibroblast cell populations were calculated by manually masking the microchannel areas on the *z*-projections of the original image stacks: these were found to be 92 ± 2% (cells in the microchannel region, i.e. HUVECs) and 89 ± 2% (cells outside the microchannel region, i.e. HDNFs) respectively. In another round of co-culture experiments, HUVECs and HNDFs showed a collective viability of 87 ± 6% (*n* = 6) after 14 d of culture.

## 4. Conclusions and future outlook

Current top–down methods to engineer microvascular structures struggle to reliably produce large volumes of microvessels with architecture mimicking that of natural capillary beds. By leveraging the simple processability, thermoresponsive solubility, and cytocompatibility of Soluplus®, we created sacrificial templates capable of patterning large volumes of interconnected microchannels within cm-thick gelatin hydrogels. Characterization of these microchannels reveals that they exhibit an architecture with parameters approaching those of natural capillary beds, including an average diameter of 26 ± 17 *μ*m, and a branch length and tortuosity closely matching those of mouse cortex capillaries. Further, we were able to culture a contiguous endothelial monolayer in said microchannels which remained reliably viable for over 21 d, exhibited cell-to-cell junction proteins at 2 weeks post-seeding, and significant barrier function at 1 week post-seeding. When our endothelialized microchannels were co-cultured with fibroblasts encapsulated within the gelatin matrix, we observed well defined niches for both cell populations and measured an overall viability of 87 ± 6% at 14 d post seeding.

While the endothelial networks exhibit an impressive degree of channel coverage and a healthy physiology, it is worth noting that not all of our channels became endothelialized. The random nature of the fabrication process inevitably yields a small number of fluidic ‘dead-ends’ which receive no flow and thus do not become seeded with ECs. While these channels have been observed across microchannel gels, they constitute a very small proportion of vessel branches and are thus not expected to have a significant impact on co-cultures vascularized by our method.

Future work will involve development of more sophisticated vessel architectures and co-culture systems. 3D-printing methods have already proven adept at creating larger vascular architecture; the integration of small fiber meshes with 3D-printed branches presents exciting opportunities for patterning multi-scale vascular architecture. In theory, any hydrogel could be applied to our fabrication method, as long as the gel can be cast at a temperature above the LCST of Soluplus®, crosslink at that temperature, and then retain the patterned channels following cooling and removal of the fibers. Fibrin is a natural polymer which meets these criteria and has been extensively used in both wound healing and scaffold for artificial vasculature [60, 61]. PEG has been modified with a range of functional groups to yield tunable mechanical properties, photosensitive side-chains, and the presentation of relevant biomolecules [62, 63]. Other potential hydrogel systems include: alginate, gelatin-methacrylate, hyaluronic acid, and poly(vinyl alcohol) to name a few [62–65]. Each of these could be integrated with our system to control additional factors in a disease model or therapeutic device.

Angiogenic sprouting models are of high interest to tissue engineers. With the integration of growth factor gradients, controlled matrix stiffness, or modified ECM materials, our microvessel production method has potential to be applied to such studies [66–68]. Further, the highly vascular tissues of the body, including the brain, heart and liver, present valuable targets for co-culture models using our artificial microvesssels.

The results presented here represent a step forward in both the patterning of microchannels and the culturing of endothelial cells within them. Our patterning method resulted in channel networks with a feature size, degree of complexity and channel density that modern top–down techniques, such as 3D bioprinting and optical fabrication, struggle to produce. By producing thick tissue cultures with endothelialized microvasculature, it is our goal to produce platforms for more physiologically relevant disease models and move towards a viable approach to making large-scale tissue replacements in regenerative medicine.

## Data Availability

All data that support the findings of this study are included within the article (and any supplementary files).
